# Giant multilocular prostatic cystadenoma in a young man: A case report from Jordan

**DOI:** 10.1002/ccr3.5517

**Published:** 2022-03-04

**Authors:** Mohammad Kh. Alzawahreh, Ala'a B. Al‐Tammemi, Mustafa I. Al‐Shalah, Ahmad Abuebeid, Zaid Manaserh, Baha'a Alhroub, Moath O. Badawi, Anas M. Abu‐Zanouneh, Maen Malkawi

**Affiliations:** ^1^ Division of Urology Department of Special Surgery Al Bashir Hospital Ministry of Health Amman Jordan; ^2^ 37599 Faculty of Medicine Department of Family and Occupational Medicine University of Debrecen Debrecen Hungary; ^3^ 37599 Doctoral School of Health Sciences University of Debrecen Debrecen Hungary; ^4^ Department of Radiology Al Bashir Hospital Ministry of Health Amman Jordan; ^5^ Department of Pathology Al Bashir Hospital Ministry of Health Amman Jordan

**Keywords:** cystadenoma, obstructive symptoms, prostate, surgical excision

## Abstract

Giant Multilocular Prostatic Cystadenoma (GMPC) is one of the rare benign tumors of the prostate. This report presents a case of a young man who has been recently diagnosed with GMPC. Our report highlights the importance of timely diagnosis and treatment, considering the overlapping symptoms with other common urinary conditions.

## Abstract 2

Despite its rarity, GMPC is a benign tumor of the prostate gland that should be taken into consideration as one of the differential diagnoses for large pelvic /retroperitoneal cystic lesions. Complete surgical excision is the treatment of choice.

## 1 INTRODUCTION

Giant multilocular prostatic cystadenoma (GMPC) is one of the rare benign tumors which originate from the prostate gland. It usually occupies the pelvis as a large pelvic mass, and most of the time, this lesion is located between the bladder and the rectum.^1^, ^2^ GMPC does not show any malignant potential or characteristics, and it does not possess invasive features. However, due to its location and the mass effect, typical symptoms for patients with this rare lesion are obstructive voiding or retention symptoms.^3^ Additionally, the patients can suffer from constipation, accounting for the mass effect on the rectum in case of large lesions. Here, we present a case of a young man from Jordan who has been recently diagnosed to have GMPC.

## 2 CASE PRESENTATION

A 20‐year‐old single, smoker, male patient, who had no significant medical or surgical history, presented to our specialized urology clinic at one of the main tertiary hospitals in Jordan with a chief complaint of intermittent obstructive and urinary retention symptoms for the last 9 months. His symptoms included urgency, urinary dribbling, weak stream, and urinary frequency of around 10 times in the daytime, as well as nocturia of 3–6 times each night. Additionally, these symptoms were associated with intermittent mild bilateral flank pain and intermittent constipation. There was no history of fever, no vomiting, and no history of urethral discharge. The patient has reported no problem with his sexual desire or erection, except for an intermittent painful ejaculation. His symptoms gradually increased with time and started to affect his daily activities with a significant negative impact on his routine life due to bothersome urinary symptoms.

Before he visited our specialized clinic, he was initially treated by a general physician as a case of urinary tract infection (UTI) with no improvement. At that time, the patient developed the first episode of urinary retention, so he was referred to the hospital and managed at ER with Foley's insertion, then he was discharged on antibiotics and was referred to an outpatient urology clinic. Two weeks later, the Foley's catheter was removed upon the patient's request and against medical advice at a primary healthcare center, and he has not visited the urology clinic as planned.

The patient was doing relatively well during the next 6 months, with intermittent tolerable urinary symptoms and constipation, until he started to have more bothersome symptoms again, which were associated with flank pain and more constipation as well. However, the patient did not seek medical help until he had the second episode of urinary retention and severe lower abdominal pain, which enforced him to visit a nearby hospital. At that time, he was managed by Foley's insertion, and around 1500 ml of clear urine was evacuated. During the same episode, a renal ultrasound was done, and the impression was suspected ureteric stone due to fullness of kidneys. Therefore, the patient was managed by tamsulosin and antibiotics, then he was discharged on the Foley's catheter along with the prescribed medications. Two weeks later, the Foley's catheter was removed for 2 days, and this has resulted in the third episode of urinary retention during which a Foley's catheter was inserted again, and the patient was referred to the specialized urology clinic at our tertiary hospital.

During physical examination at our clinic, the patient had stable vital signs with normal abdominal and genital examination. There were no palpable masses, and no flank or costovertebral angle tenderness. The scrotum and penis were within the normal secondary sexual characters. Digital rectal examination (DRE) was also inconclusive.

## 3 LABORATORY AND RADIOLOGICAL INVESTIGATIONS

**FIGURE 1:**
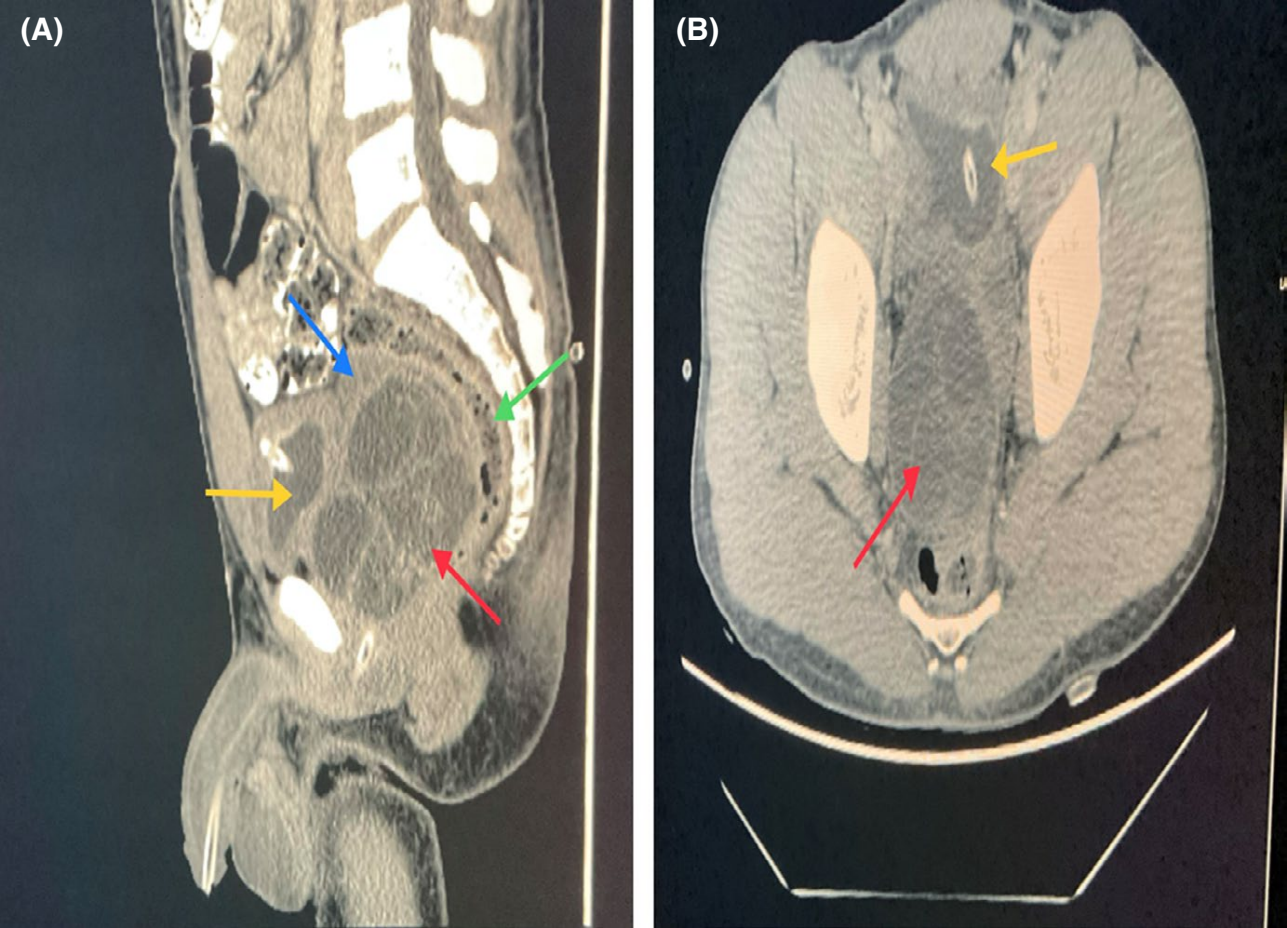
(A) Preoperative contrast‐enhanced CT scan with sagittal cuts, showing cystic lesion with enhancing septations compressing the rectum posteriorly (green arrow) and the urinary bladder anteriorly (yellow arrow) which appears under filled with Foley's catheter in situ. The seminal vesicle (blue arrow) appears compressed and pushed superiorly. (B) Preoperative contrast‐enhanced CT axial cuts, showing cystic lesion with enhancing septations (red arrow) compressing rectum posteriorly and urinary bladder anteriorly (yellow arrow) which appears under filled with Foley's catheter in situ

**FIGURE 2:**
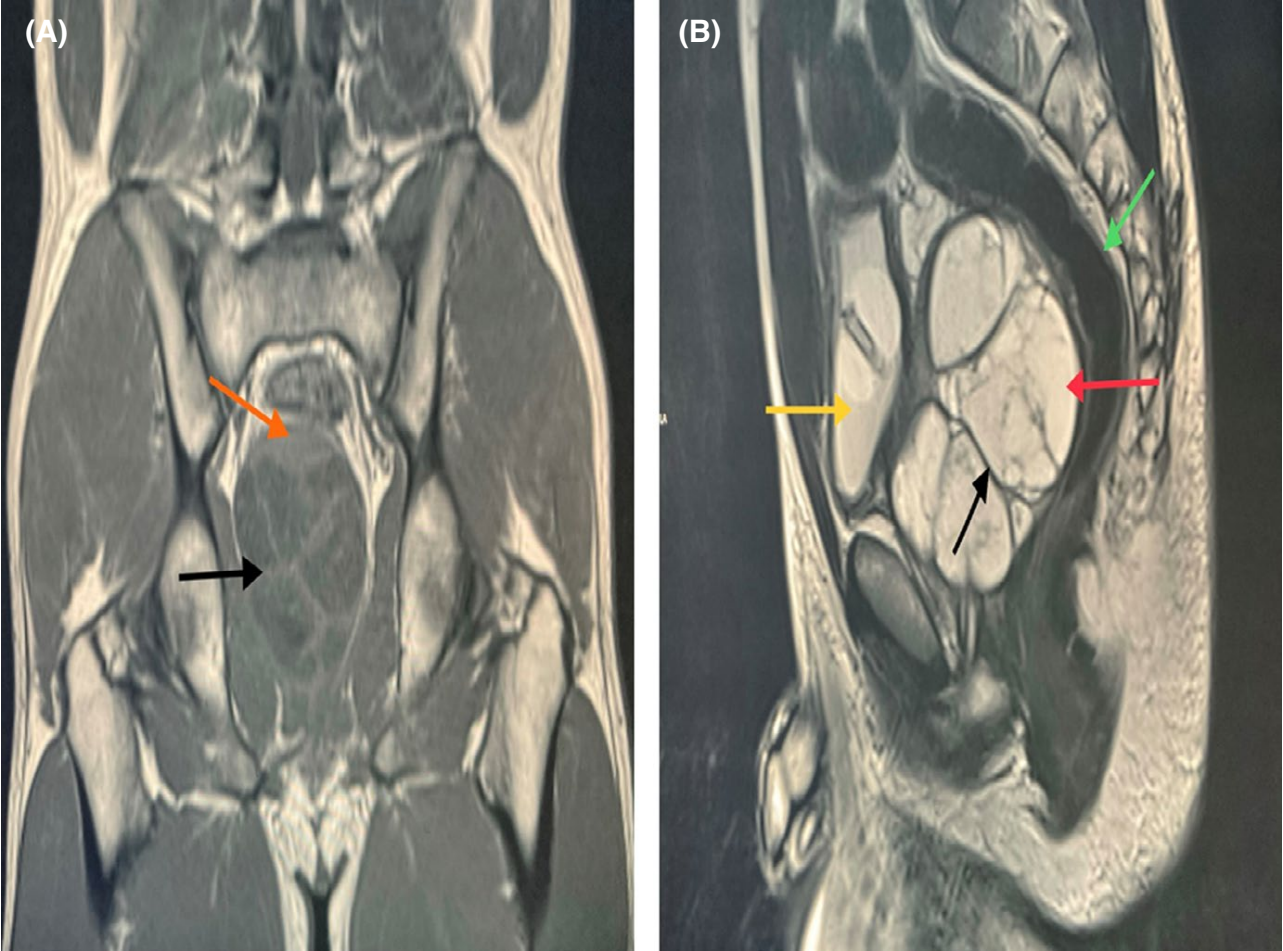
(A) Preoperative post‐contrast coronal MRI T1, showing cystic lesion with enhancing septa (black arrow) and enhancing soft tissue component (orange arrow). (B) Preoperative MRI Sagittal T2, showing large cystic lesion (red arrow) with septations (black arrow) exerting mass effect on rectum (green arrow) and urinary bladder (yellow arrow) which appeared under filled with Foley's catheter in situ

At our urology clinic, the patient has undergone laboratory investigations including complete blood count, kidney function test, urinalysis and culture, prostate‐specific antigen (PSA) level which were all found normal. Additionally, an abdominopelvic CT scan with and without contrast was done (Figure 1) and revealed a diffuse wall thickening of the bladder, and a large well‐circumscribed multilocular cystic mass with multiple septations and soft tissue component along its left anterolateral aspect seen in the expected site of the prostate, right to the midline, with Foley's catheter passing intimately to the left lateral aspect of the lesion. There were no ascites. Both kidneys, liver, spleen, and pancreas were unremarkable. The radiologist advised proceeding with an MRI study. Later, the MRI revealed a large, well‐defined, multiloculated prostatic lesion, which appeared hypointense on T1, hyperintense on T2 with no evidence of diffusion restriction. Post‐contrast MRI showed evidence of enhancing soft tissue components with no evidence of gross invasion. The mass effect of adjacent structures was also noted, deviating the prostatic urethra to the left. There was no evidence of seminal vesicle invasion, no free fluid in the pelvis, no significant pelvic lymph node enlargement (Figure 2). The MRI findings were suggestive of prostatic cystadenoma, cystic prostatic carcinoma, or a hydatid cyst. Consequently, the definitive diagnosis requires excision with histopathological correlation.

## 4 SURGICAL TREATMENT

In the beginning, the patient underwent diagnostic urethro‐cystoscopy under lithotomy position to assess the urethra, prostate, and bladder, and the process was challenging. We noticed a hyperemic prostatic urethra with some blood clots and hypervascularity, narrowing of prostatic part of urethra mostly from the compressing effect of the cyst. Also, there was an inability to identify ureteric orifices due to bladder trabeculation and hyperemic lining bladder mucosa.

**FIGURE 3:**
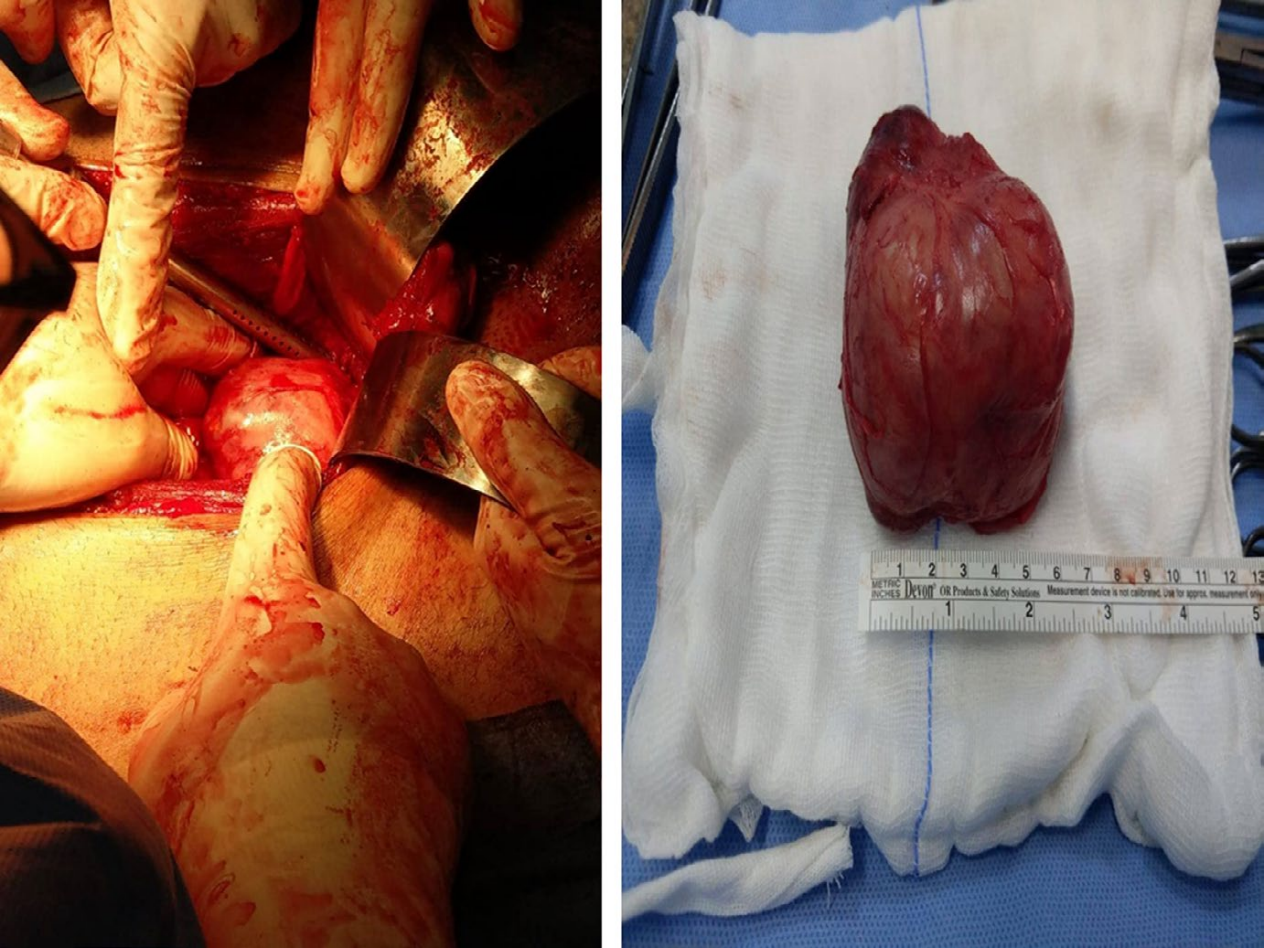
Intraoperative image of the surgical exploration (left) and the excised mass (right)

After that, a Foley's catheter was inserted, and the patient was turned to supine position, followed by a lower midline incision, and the exploration process was started. Upon exploration, a deep large pelvis mass was identified which was attached to the bladder, with a most probable origin from the prostate gland. A complete dissection was done, with securing the vessels, adjacent organs, and structures. After dissection, a complete mass resection was done (Figure 3). Grossly, the mass was multi‐cystic and measured about 8.0 × 7.0 × 3.0 cm (Figure 3). After excision of the mass, three drains were inserted (pelvic, suprapubic, and bladder).

## 5 HISTOPATHOLOGY

**FIGURE 4:**
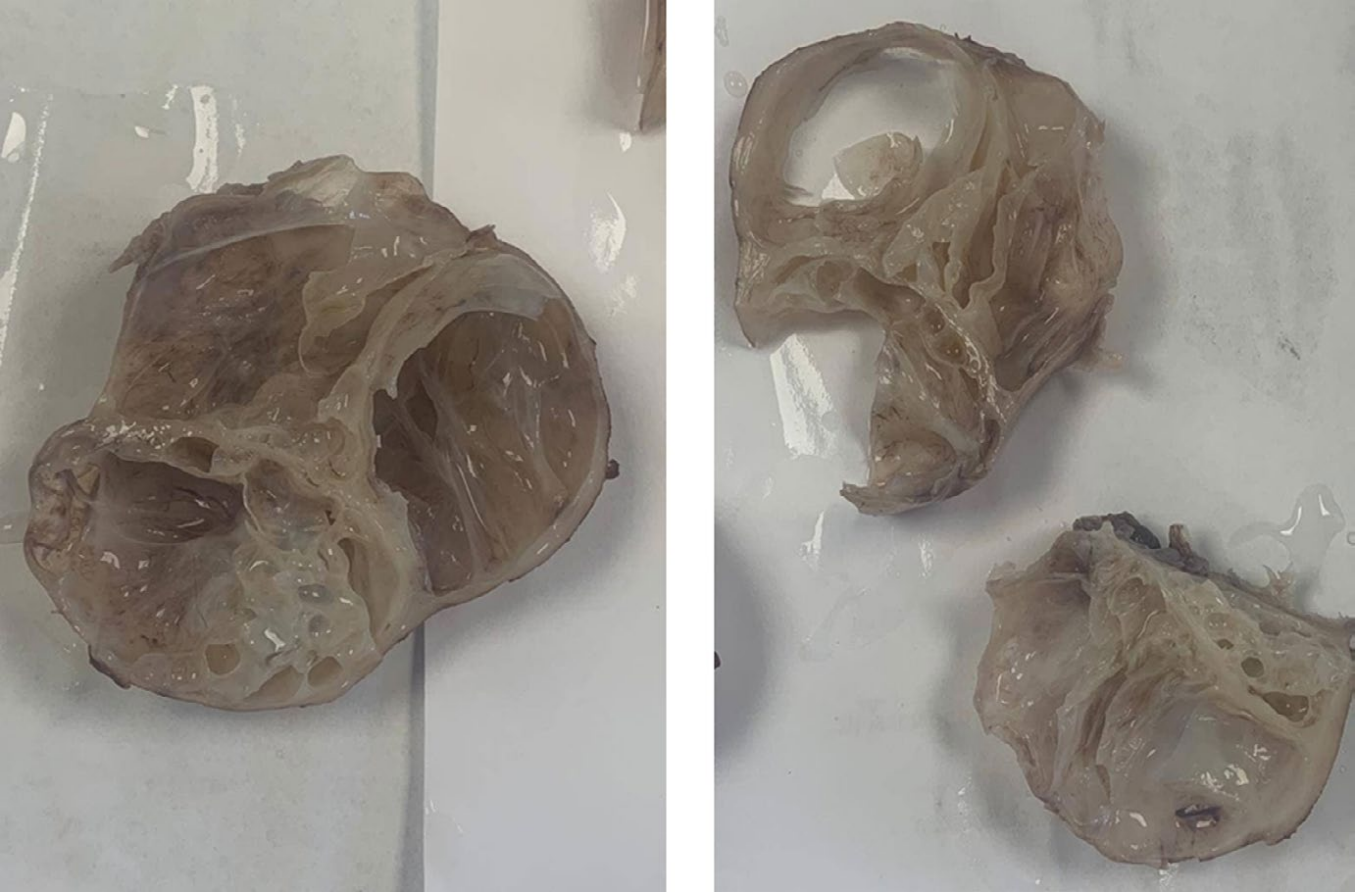
Cuts for gross pathologic specimen shows multilocular cystadenoma of the prostate

**FIGURE 5:**
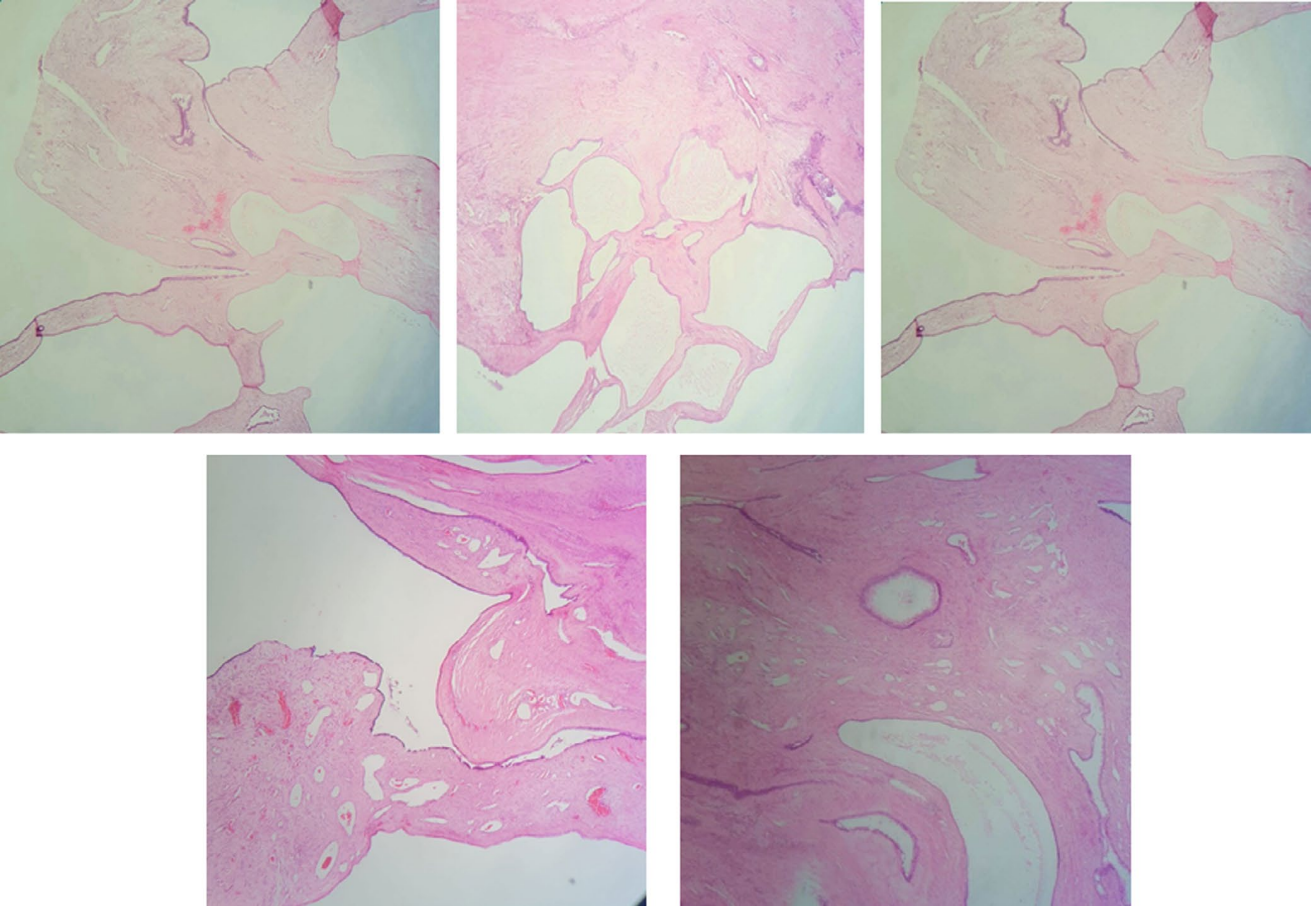
Histological examination of the resected specimen (H&E stain). Cystic wall containing numerous acini and dilated glandular structures. Glands and cysts are lined by cuboidal to low columnar epithelial cells

**FIGURE 6:**
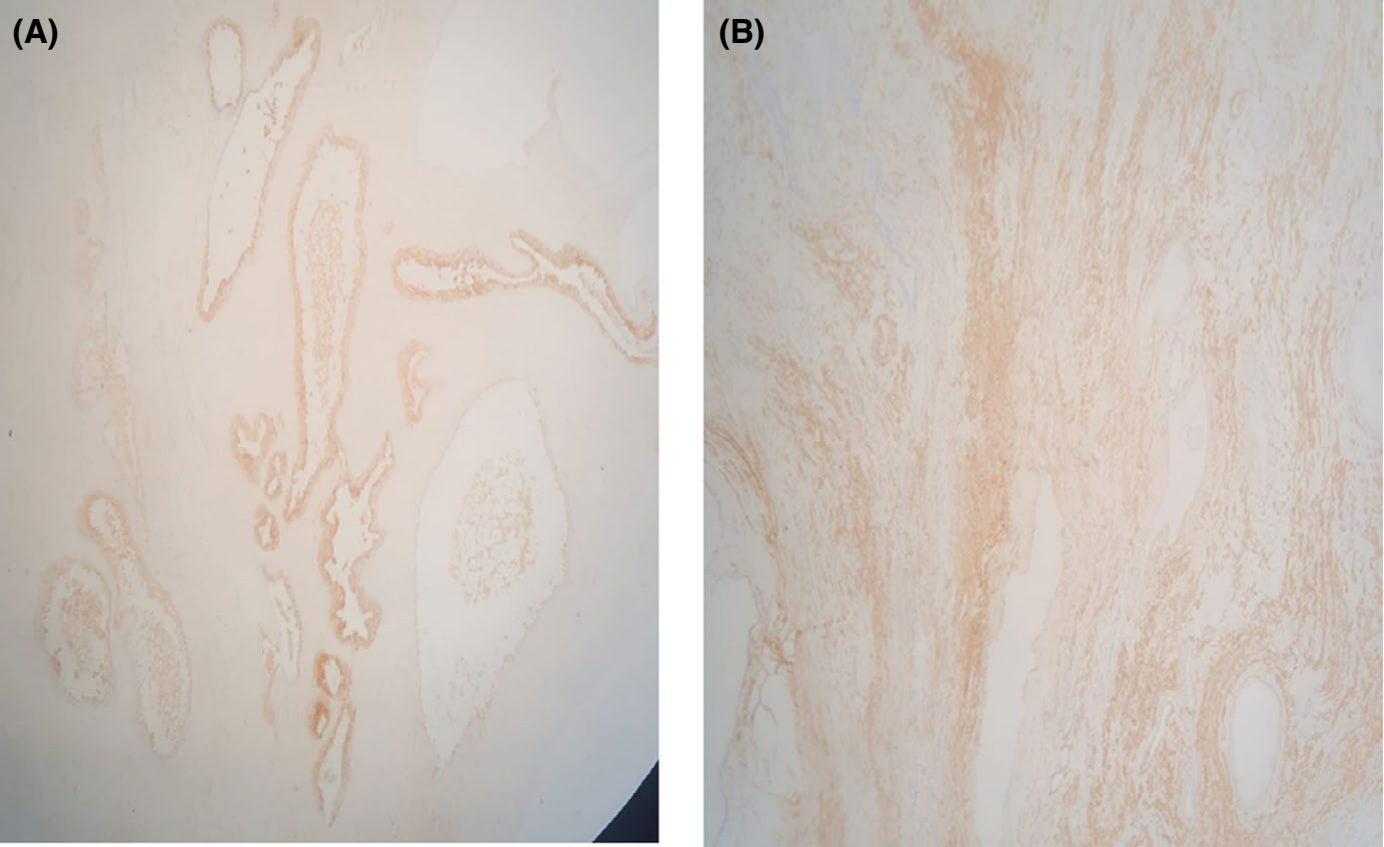
(A) PSA stain: Immunohistochemical (IHC) stain shows diffuse positivity of the gland (positive PSA stain). (B) Immunohistochemical (IHC) stain shows diffuse cytoplasmic positivity of the smooth muscle cells (positive smooth muscle actin [SMA] stain)

**FIGURE 7:**
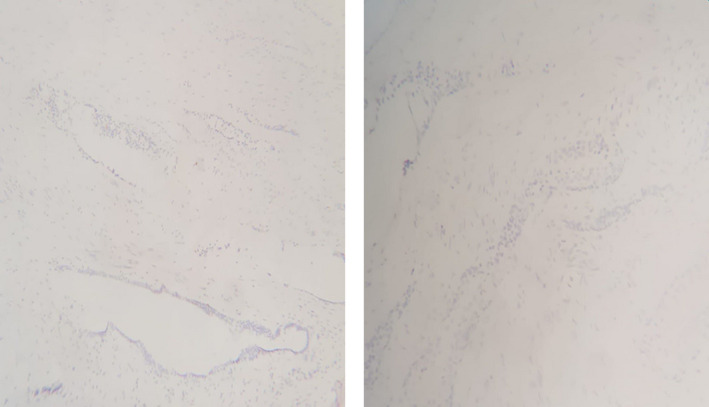
Immunohistochemical (IHC) stain shows negativity for both WT‐1 and HMWK (high molecular weight cytokeratin)

The histological examination of the resected mass showed multilocular prostatic cysts, some were massively enlarged. The cysts were lined by cuboidal to low columnar type epithelium. Another flattened epithelium of prostatic type was also confirmed by positive cytoplasmic staining for PSA and Pankeratin immune stains. The surrounding fibromuscular stroma was unremarkable, and there was no evidence of malignancy. For the detailed histopathological assessment, see Figures 4, 5, 6, 7.

## 6 POSTOPERATIVE FOLLOW‐UP

**FIGURE 8:**
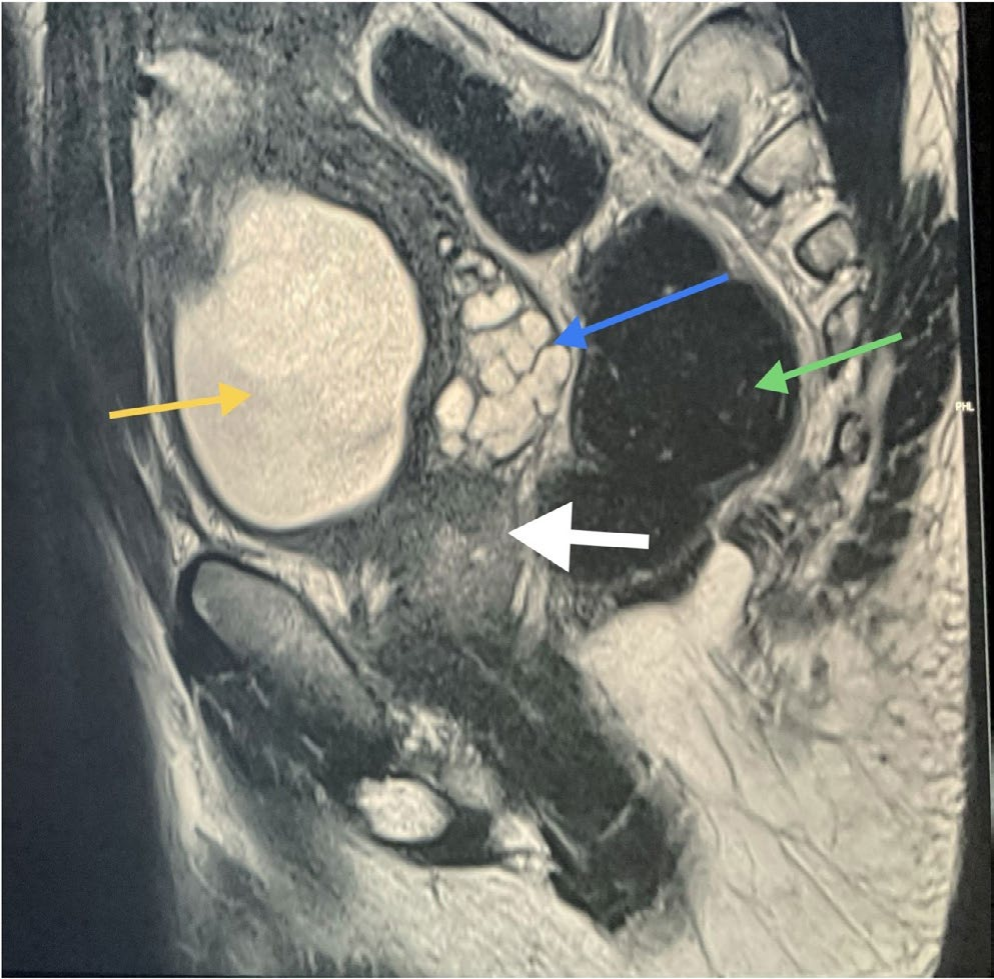
Postoperative MRI Sagittal T2. White arrow (site of resected mass), Yellow arrow (Urinary Bladder) Green arrow (Rectum). Blue arrow (Seminal vesicle)

The patient had a smooth postoperative period. He was discharged after 7 days of admission, and the discharge medications included simple analgesics. A cystogram was done 3 weeks post‐operatively and showed no extravasation of urine from the bladder. An MRI study was conducted 1 month after the surgical resection and revealed a recent complete mass resection (Figure 8). Also, 1 month following the surgery, the patient reported normal erection and ejaculation, along with normal bowel motions and normal urination with no voiding symptoms. Three months postoperatively, the patient was doing well with no complaints.

## 7 DISCUSSION

According to the literature, there are fewer than 30 cases of GMPC that have been reported globally since 1991,^1^, ^4^ and to the best of our knowledge, this is the first reported case from Jordan. The age of patients with GMPC could be as young as 16 years old up to 80 years old, as reported in previous case reports.^2^ In our case, the patient was 20 years old. Patients with GMPC could be falsely diagnosed to have giant benign prostatic hyperplasia (BPH), and this is due to the shared obstructive and irritative urinary symptoms between the two clinical entities.^1^, ^2^, ^3^, ^5^ Less common symptoms of GMPC include palpable abdominal mass, constipation, and obstructive azoospermia.^2^, ^5^ In our case, the patient has also suffered from constipation which was mostly attributed to the mass effect on the rectum caused by the prostatic lesion. Physical examination may find some signs of obstruction (distended bladder, fecal impaction), and an enlargement of the prostate is noticed by DRE. PSA value may or may not be elevated, as in our case the PSA level was within normal range.

The diagnosis of GMPC was challenging, especially before the advancement in various imaging modalities. Therefore, radiological studies play a crucial role in preoperative diagnosis, especially in identifying the lesion and its relationship to the adjacent structures and organs. Therefore, advanced imaging techniques such as MRI may provide additional information for both further characterization, extent of local invasion and presurgical planning.^3^

In general, cystic prostatic lesions can be divided according to their location into median, paramedian, or lateral.^3^ Besides the location assessment, further classification can be done according to the relationship with the prostatic urethra, as either prostatic or periprostatic.^3^ The majority of prostatic cystic lesions will include utricle cysts, Müllerian cysts, ejaculatory cysts, and seminal gland cysts, with the two former being commonly median and the last two lesions being more commonly lateral. On the other hand, there are other differential diagnoses that resemble GMPC, which may include the phylloid variant of atypical prostatic hyperplasia, prostatic leiomyoma, prostatic sarcoma, cystic prostatic carcinoma, pelvic mesothelioma, lymphangioma, peritoneal inclusion cyst, and teratomas. Moreover there is infectious reason for such a growth which usually occurs in patients with clinical signs and symptoms of infection (hydatic cysts, echinococcosis, or cavitary prostatitis with or without abscesses)^1^, ^2^, ^3^, ^5^, ^6^, ^7^

The definitive diagnosis of GMPC is confirmed with histologic examination of the resected mass. Histological examination usually shows dilated/cystic prostatic gland, but with the typical lining of columnar and cuboidal cells, pale cytoplasm, and the nucleus is in the base of the cells, in a background of hypocellular fibrous stroma. Regarding the results of immunohistochemical staining, as in our case, reports indicated that multilocular prostatic cystadenoma was positive for PSA, which confirms that the mass originates from the prostate.

Most of the reported cases in the literature were managed by complete surgical excision, which is curative with few reported recurrences have been encountered, especially with incomplete resection^1^, ^4^. However, gonadotropin‐releasing hormone antagonist (GnRH) was found to be effective in the treatment of recurrent GMPC.^7^ Additionally, various complications were reported in the literature during complete excision of GMPC lesions such as pelvic abscess due to urine leakage from the prostatic urethra,^1^ accidental injury of the anal canal which required rectoraphy with protective colostomy,^6^ and an accidental bladder injury which was repaired and rectal leak during dissection which was sutured and protected by a left iliac colostomy.^8^ In our case, we had one complication as well. During dissection of the mass, we accidently identified two small bladder wounds which were successfully sutured, and suprapubic catheter was inserted.

As most of the reported cases in the literature had complete recovery without recurrence of urinary or sexual symptoms, and this was noticed as well in our case. In conclusion, and despite its rarity, GMPC is a benign tumor of the prostate gland that should be taken into consideration as one of the differential diagnoses for large pelvic /retroperitoneal cystic lesions. As GMPC is still difficult to diagnose due to its relative rarity, but every clinician should be aware of this condition when a retro‐vesical cystic lesion fills the pelvis in a male patient with voiding or defecation symptoms. Surgery is often necessary in most of the cases and the excision must be complete to avoid recurrence and regrowth of masses.

## CONFLICT OF INTEREST

The authors declare that they have no conflict of interest.

## AUTHOR CONTRIBUTIONS

MKA and ABA involved in conceptualization and writing—original draft preparation; ABA involved in supervision; MKA, ABA, AA, MIA, ZM, BA, MOB, AMA, and MM involved in literature review; and: AA, MIA, ZM, BA, MOB, AMA, and MM involved in writing—critical review and editing. All authors have read and approved the submitted manuscript.

## ETHICAL APPROVAL

This was conducted following the ethical standards of the World Medical Association Declaration of Helsinki. The patient has given his written informed consent.

## CONSENT

Yes.

## Data Availability

All the data are reported in the manuscript.
